# Distinct colonization patterns and cDNA-AFLP transcriptome profiles in compatible and incompatible interactions between melon and different races of *Fusarium oxysporum *f. sp. *melonis*

**DOI:** 10.1186/1471-2164-12-122

**Published:** 2011-02-21

**Authors:** Sara Sestili, Annalisa Polverari, Laura Luongo, Alberto Ferrarini, Michele Scotton, Jamshaid Hussain, Massimo Delledonne, Nadia Ficcadenti, Alessandra Belisario

**Affiliations:** 1 Agricultural Research Council (CRA), Research Unit for Vegetable Crop in Central Areas, Via Salaria 1, 63030 Monsampolo del Tronto (AP), Italy; 2 Department of Biotechnology, University of Verona, Strada Le Grazie 15, 37134 Verona, Italy; 3 Agricultural Research Council (CRA), Plant Pathology Research Center, Via C.G. Bertero 22, 00156 Roma, Italy; 4 Department of Environmental Agronomy and Crop Production, University of Padova, Viale dell'Università 16, 35020 Legnaro, Padova, Italy

## Abstract

**Background:**

*Fusarium oxysporum *f. sp. *melonis *Snyd. & Hans. (FOM) causes Fusarium wilt, the most important infectious disease of melon (*Cucumis melo *L.). The four known races of this pathogen can be distinguished only by infection on appropriate cultivars. No molecular tools are available that can discriminate among the races, and the molecular basis of compatibility and disease progression are poorly understood. Resistance to races 1 and 2 is controlled by a single dominant gene, whereas only partial polygenic resistance to race 1,2 has been described. We carried out a large-scale cDNA-AFLP analysis to identify host genes potentially related to resistance and susceptibility as well as fungal genes associated with the infection process. At the same time, a systematic reisolation procedure on infected stems allowed us to monitor fungal colonization in compatible and incompatible host-pathogen combinations.

**Results:**

Melon plants (cv. Charentais *Fom-2*), which are susceptible to race 1,2 and resistant to race 1, were artificially infected with a race 1 strain of FOM or one of two race 1,2 w strains. Host colonization of stems was assessed at 1, 2, 4, 8, 14, 16, 18 and 21 days post inoculation (dpi), and the fungus was reisolated from infected plants. Markedly different colonization patterns were observed in compatible and incompatible host-pathogen combinations. Five time points from the symptomless early stage (2 dpi) to obvious wilting symptoms (21 dpi) were considered for cDNA-AFLP analysis. After successful sequencing of 627 transcript-derived fragments (TDFs) differentially expressed in infected plants, homology searching retrieved 305 melon transcripts, 195 FOM transcripts expressed *in planta *and 127 orphan TDFs. RNA samples from FOM colonies of the three strains grown *in vitro *were also included in the analysis to facilitate the detection of *in planta*-specific transcripts and to identify TDFs differentially expressed among races/strains.

**Conclusion:**

Our data suggest that resistance against FOM in melon involves only limited transcriptional changes, and that wilting symptoms could derive, at least partially, from an active plant response.

We discuss the pathogen-derived transcripts expressed *in planta *during the infection process and potentially related to virulence functions, as well as transcripts that are differentially expressed between the two FOM races grown *in vitro*. These transcripts provide candidate sequences that can be further tested for their ability to distinguish between races.

Sequence data from this article have been deposited in GenBank, Accession Numbers: HO867279-HO867981.

## Background

*Fusarium oxysporum *Schltdl.:Fr. is an anamorphic fungal soil-borne facultative parasite present in soil and on organic substrates worldwide. The species includes non-pathogenic and pathogenic strains, the latter causing vascular wilt and root rot on many economically important crops. Pathogenic *F. oxysporum *strains have been subdivided into over 70 different host-specific forms (*formae speciales *or f. sp.) which are morphologically indistinguishable and represent intra-specific groups of strains with similar or identical host range [1,2]. The identification of pathogenic *F. oxysporum *isolates is traditionally based on pathogenicity testing, which is time consuming and laborious. A *forma specialis *can be further subdivided into races on the basis of characteristic virulence patterns on differential host cultivars [3].

Among the eight *formae speciales *that attack cucurbits, only *F. oxysporum *f. sp. *melonis *Snyder & Hans. (FOM) is specific to melon (*Cucumis melo *L.) and it is responsible for the most important infectious disease in this fruit species [4]. Four races of the pathogen (0, 1, 2 and 1,2) have been defined according to the host resistance genes overcome by variants of the pathogen [5]. Race 1,2 is further subdivided into race 1,2 y, which causes yellowing, and race1,2 w, which causes wilting. Race 0 induces disease on melon genotypes that lack FOM resistance genes. Two dominant, independently-inherited resistance (R) genes (*Fom-1 *and *Fom-2*) provide resistance to races 0 and 2, and races 0 and 1, respectively [5]. The presence of both genes confers high resistance to races 0, 1, and 2 [6]. Another gene, *Fom-3*, has been reported to confer resistance to races 0 and 2 in cultivar Perlita FR, but there are conflicting data suggesting allelism with *Fom-1 *[7]. Resistance to race 1,2 is complex and appears to be controlled by multiple recessive genes. Partial resistance was found in several Far-Eastern lines such as Ogon 9, and was introgressed into the cultivar 'Isabelle' [8] from which the two doubled-haploid resistant lines Nad-1 and Nad-2 were derived [9]. Perchepied and Pitrat [8] estimated that 4-14 genes were involved in resistance against FOM race 1,2, confirming its polygenic nature. QTL analysis revealed nine loci linked to this trait in melon [10]. More recently, Herman and Perl-Treves [11] found that two complementary recessive genes in the genotype BIZ are required to confer full resistance to race 1,2. Furthermore, a major recessive QTL for resistance was located and linked to a locus controlling fruit netting [12].

Wilting symptoms and plant death caused by FOM (particularly by race 1,2) can be devastating, with losses as high as 100% [13,9,14]. Once introduced into the field, FOM can persist even after rotation with non-host crops, due to the production of chlamydospores (resting and durable spores) and its ability to colonize crop residues and roots of most crops grown in rotation [15]. Effective control can be achieved only through host resistance. Although many *Fusarium *species can penetrate into the cortical tissue of roots, only host-specific strains can penetrate the vascular elements by mycelial growth and the formation of microconidia, transported in the sap stream [2]. Unfortunately, molecular discrimination of *F. oxysporum *isolates is seriously complicated by the polyphyletic nature of many *formae speciales*, and isolates belonging to different *formae speciales *may be more related than isolates belonging to the same *forma specialis *[16]. Ideally, it would be possible to distinguish *F. oxysporum *strains based on DNA sequences directly related to (host-specific) pathogenicity or non-pathogenicity [16].

Penetration of host roots is an active process, although it may be accelerated by wounding. The progress of the infection for xylem-colonizing *F. oxysporum *strains has been documented in studies using green fluorescent protein (GFP) as a marker, mainly in melon [17,2,12,20] but also in *Arabidopsis *and tomato [21,22]. Wilting is the outcome of a combination of regulated host-pathogen activities beginning with recognition of the host root, followed by differentiation and attachment of an appressorium-like structure, penetration of root cortex to access the vascular tissue, adaptation to the hostile plant environment, hyphal proliferation and production of microconidia within the xylem vessels, and finally the secretion of small molecules such as peptides or toxins [18,2]. The host responds with molecular defenses and with the production of defence structures including gels, gums, and tyloses, and vessels crashing by proliferation of adjacent parenchyma cells [23,24]. Understanding the molecular aspects of the infection process could shed light on the mechanisms and genes involved in the signal cascades associated with resistance and susceptibility. The response to *F. oxysporum*, as a vascular pathogen, has predominantly been characterized in the host/pathogen binomial tomato/*F. oxysporum *f. sp. *lycopersici *which has become a model system for the molecular basis of disease resistance and susceptibility [25]. Some resistance mechanisms have been determined by gene silencing or insertional mutagenesis [18,2,26,27]. Understanding susceptibility/resistance in melon would facilitate the development of new control strategies and the identification of pathogen and host factors required for resistance responses and/or disease progression.

Changes in host and pathogen steady state mRNA levels during a fungal infection can provide a valuable readout of the molecular processes underlying resistance and susceptibility [28]. DNA microarrays are traditionally the standard tool for genome-wide expression analysis, although next-generation sequencing technologies are emerging as a robust alternative, but in both cases large collections of known transcript sequences must already be available [29]. In contrast, cDNA-AFLP remains the method of choice where the focus is gene discovery, particularly when dealing with plant-microbe interactions and seeking to identify transcripts from both interacting partners [30,31].

Here we describe the identification of differentially expressed transcripts in the binomial interaction between melon and FOM. The cultivar Charentais *Fom-2 *was chosen as the host genotype since it is susceptible to FOM race 1,2 but resistant to race 1, thus providing the opportunity to investigate both compatible and an incompatible interactions in the same genetic background. We infected plants with FOM strain ISPaVe1070 (race 1) and strains ISPaVe1018 and ISPaVe1083 (race 1,2 w). The race 1,2 w strains are both highly virulent, but only ISPaVe1083 commonly induces necrosis at the collar level. These strains were chosen to identify possible differences in gene expression between isolates differing in their aggressiveness. Host colonization in stems was assessed at 1, 2, 4, 8, 14, 16, 18 and 21 days post inoculation (dpi), and the fungal strains were reisolated from infected plants. We observed markedly different colonization patterns when comparing compatible and incompatible host-pathogen combinations. Five time points (0, 2, 4, 8 and 21 days) from the symptomless early stage (2 dpi) to obvious wilting symptoms (21 dpi) were considered for cDNA-AFLP analysis to identify both early signaling events occurring in the plant, and plant or fungal genes possibly involved in symptom development. Because of the increase in fungal mass at late time points, the analysis was expected to identify a large number of fungal transcripts expressed *in planta*, particularly at 21 dpi, when wilting symptoms in the compatible interaction are obvious. RNA from colonies of the three strains grown *in vitro *was also included in the analysis to help detect FOM transcripts specifically expressed *in planta *and to identify transcript-derived fragments (TDFs) that are differentially expressed among races/strains.

## Results

### Melon colonization by *Fusarium oxysporum *f. sp. melonis races 1 and 1,2

**Figure 1:**
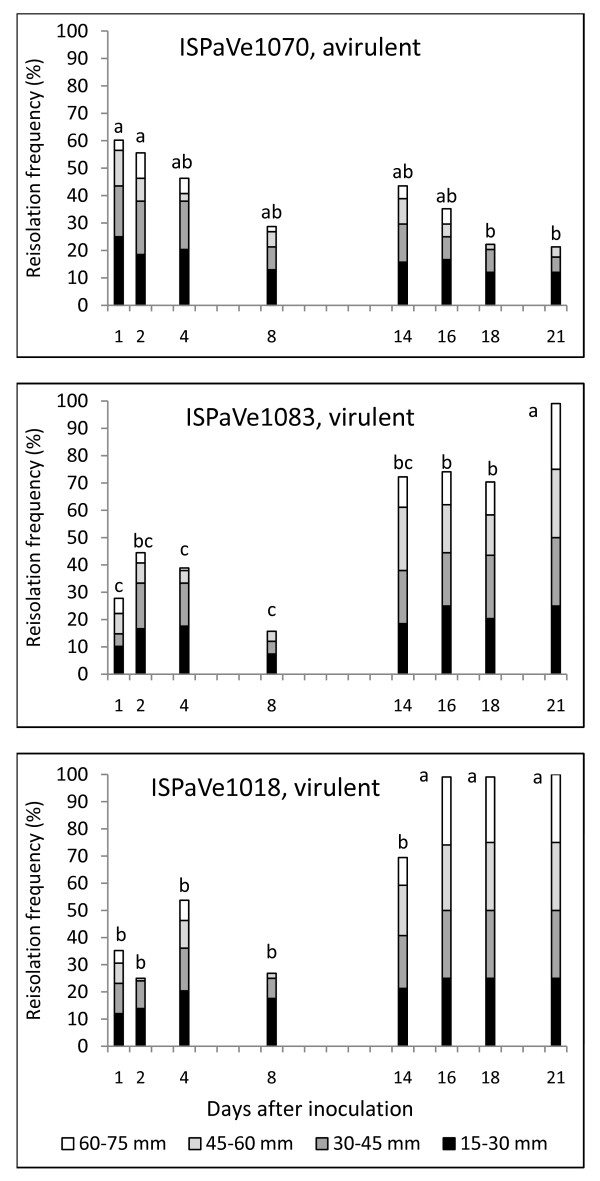
**Vascular colonization patterns of *Fusarium oxysporum *f. sp. *melonis *(ISPaVe1070 race1, and ISPaVe1018 and 1083 race 1,2 w) on Charentais *Fom-2 *within a period of 21 days after inoculation**. The frequency of reisolation (%) is based on fungal colonies obtained from fragments 5 mm in length, cut along the stem to a maximum height of 75 mm. The values of the four sections of each column were calculated by summing the numbers of reisolations for each of the four height classes (15-30 mm, 30-45 mm, 45-60 mm and 60-75 mm). Each section of the column shows the reisolation frequency of the corresponding height class. Its value was calculated by re-scaling the frequency of reisolation found in the stem fragments of the class to the range 0-25 (0 = no presence; 25, presence in all fragments of the class), so that when the fungus was reisolated in all fragments of all classes the frequency is 100%. Letters above the columns show the results of the pairwise contrasts between the percentage of the frequencies of fungal reisolation in all stem fragments (the upper value of each column) performed within the non parametric ANOVA: values without common letters differ at the nominal P level ≤ 0.0018 (actual P ≤ 0.05 level).

**Figure 2:**
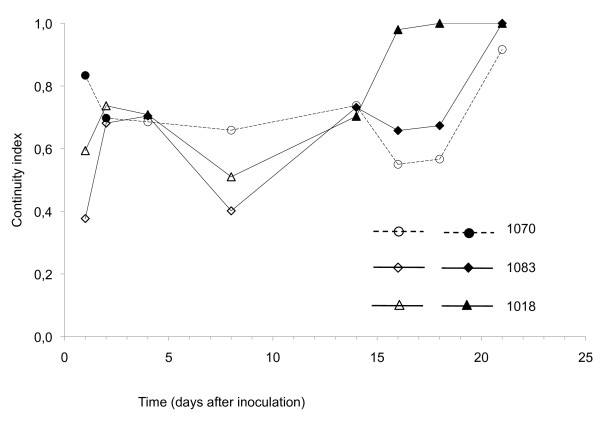
**Distribution of the fungus along the stem expressed as continuity index, within a period of 21 days after inoculation**. The index was determined for each plant by considering the presence (1) or absence (0) of the fungus in pairs of adjacent stem sections. The symbols are filled when the percentage of reisolation of the fungus is greater than 50%, and empty when the percentage is less than 50%.

The frequency of reisolations along the stem of inoculated melon plants at eight time points (1, 2, 4, 8, 14, 16, 18 and 21 dpi) is shown for the three strains of FOM in Figure 1. Both race 1 and race 1,2 were recovered from the stems of inoculated plants, irrespective of the compatibility of the host-pathogen combination, but the strains differed in the speed and extent of colonization. Avirulent strain ISPaVe1070 (race 1) achieved a more rapid and continuous colonization of the stem compared to strains ISPaVe1018 and ISPaVe1083 (race 1,2) at 1 and 2 dpi. However, by 16 dpi, the highest stem level from which the fungus could be recovered was no longer dependent on the race. All strains reached a height of 75 mm, although ISPaVe1018 did so with the greatest frequency. By 18 and 21 dpi, when symptoms of the virulent strains were obvious on all plants, both race 1,2 strains could be reisolated all along the stems, although ISPaVe1018 was faster and more continuous than ISPaVe1083. Conversely, the avirulent strain was never recovered from the highest section of the stems (60-75 mm) from 18 dpi and onwards. A continuity index was established for each plant by considering the presence (1) or absence (0) of the fungus in adjacent pairs of stem sections. Generally, the distribution of fungus along the stem was discontinuous at the early time points, although more continuity was shown by race 1 (Figure 2). A peculiar pattern was shown at 8 dpi because the highest section allowing successful reisolation was lower than at 1-4 dpi (Figure 1), and the pathogen distribution was still discontinuous for all races (Figure 2). From 14 dpi onwards, colonization along the stem differed significantly between the virulent and avirulent strains, and the more extensive colonization shown by the race 1,2 strains was coupled with the appearance of obvious symptoms. In the late phase (14-21 dpi), continuous distribution was observed for all three strains, but for race-specific reasons (Figure 2). Both virulent strains were continuously distributed along the entire stem length, whereas the avirulent strain was continuously absent from the highest section of the stem, and continuously present in the lower sections (Figure 1). Plants inoculated with race 1 remained symptomless until the end of the experiment, although the pathogen could still be reisolated. The fungus was never reisolated from uninoculated plants. All three strains could be reisolated from the stem base regardless of the time point.

### cDNA-AFLP analysis

We carried out a cDNA-AFLP analysis on RNA samples from both healthy and infected melon plants to identify differentially-expressed transcripts putatively associated with the infection process and resistance response. RNA samples from the three fungal strains grown *in vitro *were also included in the analysis, first to help identify fungal transcripts expressed specifically *in planta *and second to identify fungal genes differentially expressed *in vitro *among the three strains. Because the fungus could be reisolated from infected stems starting from 1 dpi in all interactions (Figure 1), samples of infected plants were collected for cDNA-AFLP analysis at 2, 4, 8 and 21 dpi. These time points were intended to take into account the early stages of infection, but also to allow the detection of pathogen transcripts when infection was well established and the mycelia produced at the late stage were abundant. RNA was also collected from uninfected plants as a control. The expression patterns of approximately 7000 transcripts were monitored with 128 different *Bst*YI +1/*Mse*I +2 primer combinations for selective amplification. For each primer combination, 55-75 transcript derived fragments (TDFs) were visualized as bands, varying in size from 20 to ~500 bp. The same average number of bands per lane was obtained from both melon and FOM colony samples (data not shown).

### *Detection of differentially expressed transcripts *in planta

All bands were scored visually, and only those showing a 2-fold or more difference in intensity compared to uninfected controls were selected for further analysis. All bands were assigned a positive or negative score from -3 to 3 considering as zero the intensity of the corresponding band in the control lane (0: no change; 1 = ~2-fold change; 2 = 3-4 fold change; 3 = more than 4-fold change). We detected a total of 1185 differentially-expressed TDFs.

**Figure 3:**
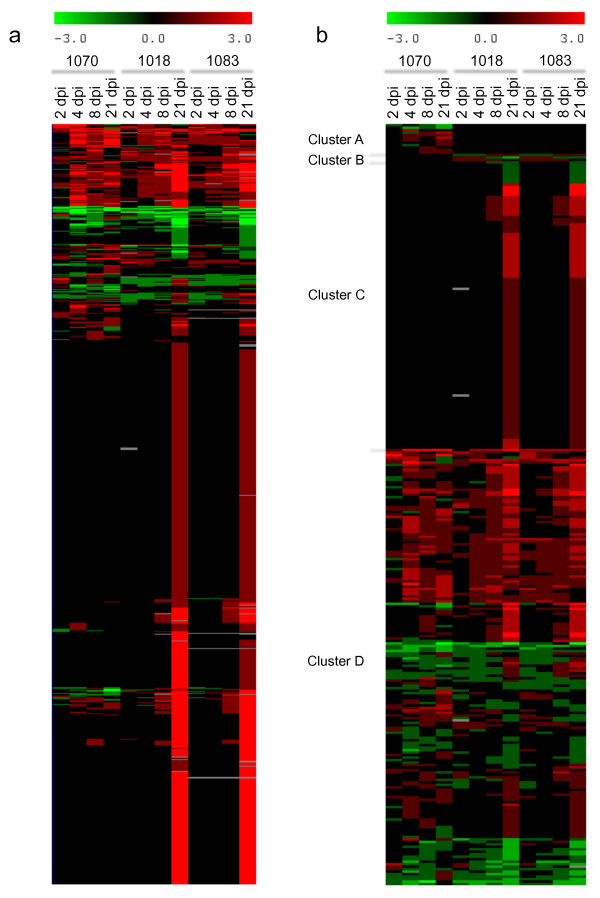
**Cluster analysis of expression profiles of melon and fungal transcripts displayed by cDNA-AFLP**. (a) Clustering of all the cDNA-AFLP profiles corresponding to differentially expressed TDFs selected for re-amplification and sequencing, possibly belonging to either the plant or the pathogen. (b) Clustering of the cDNA-AFLP profiles corresponding to differentially expressed melon TDFs.

The same scores were used to cluster profiles of differentially-expressed TDFs derived from infected melon stems. As shown in Figure 3a, FOM infection increased the abundance of a large number of mRNAs, especially in stems infected by the two race 1,2 strains at the very late phase (21 dpi), with a similar pattern of transcriptional changes. More limited changes were observed at the other time points, and also in the incompatible interaction with race 1.

A total of 970 bands with differential expression profiles in comparison to uninfected samples in at least one interaction were excised from the gels, eluted, and re-amplified with the appropriate cDNA-AFLP primers. Direct sequencing of 882 cDNA fragments yielded 627 products that could be used to screen public databases for homologous sequences, considering as significant alignments with an E < 10^-5^.

From the BLAST analysis, 305 sequences were identified as melon transcripts (Additional File 1). Among those, 111 were found to be similar to expressed melon sequences in the vs 4.0 Melon Unigene database http://www.icugi.org[32] and 115 TDFs were homologous to known plant sequences in the UNIPROT [33] or NCBI [34] databases and were therefore also considered to be derived from melon. We decided another 79 sequences with no database matches were also derived from melon because a band of identical size was present in the uninfected melon sample. A total of 195 TDFs were found to be homologous to known *Fusarium *spp. sequences and were classed as FOM genes expressed *in planta *during the infection process (Additional File 2). Most of these (193) were derived from bands detected in samples infected with the virulent race 1,2 strains at 21 dpi, when the fungus had already extensively colonized the stem.

Another 127 fragments with no matches could not be assigned to either the plant or the fungus and therefore were considered as orphan TDFs. Because most of the orphan TDFs were identified at the final time point, and only in the compatible interactions, many of them are likely to represent additional fungal transcripts that currently lack functional annotations.

### Expression patterns and clustering of melon TDFs

**Figure 4:**
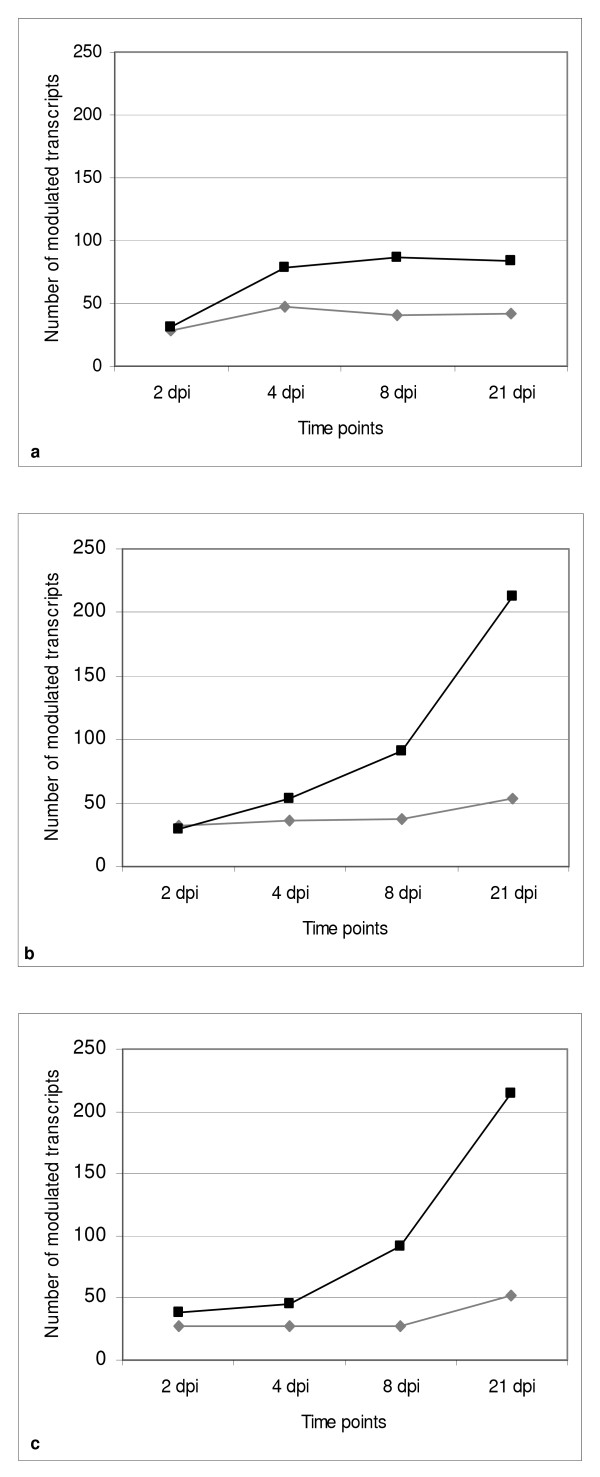
**Total number of melon genes modulated in each interaction**. Number of melon transcripts upregulated (black squares) or downregulated (gray rhombs) in the interaction with FOM race 1 strain ISPaVe1070 (a), or with FOM race 1,2 strain ISPaVe1018 (b) or strain ISPaVe1083 (c).

A numerical overview of the differences between incompatible and compatible interactions showing the total number of differentially-expressed melon genes at each time point and in each interaction is provided in Figure 4. At 2 dpi, it is clear that the response to both race 1 and race1,2 involves the same number of genes in all interactions, but differences emerge between the races at later time points. In the incompatible interaction, the number of modulated genes stabilizes at ~125, with a core of 54 genes that remain modulated in a coherent way (induced or repressed) until the end of the experiment (Additional File 3). Conversely, in the compatible interactions, the number of modulated genes increases similarly for both strains, with a peak of induction at 21 dpi.

To further characterize host plant responses towards the two races, we clustered the 305 modulated melon genes in four groups corresponding to: A) 11 melon sequences modulated solely during the incompatible interaction (Charentais *Fom-2 *inoculated with race 1); B) 3 melon sequences differentially modulated specifically in the compatible interactions at all time points in the experiment; C) 115 melon sequences modulated specifically in the compatible interactions and only at late time points (8 and 21 dpi); and D) 176 melon sequences repressed or induced at different stages in both the compatible and incompatible interactions (Additional File 1). These groups correspond to the clusters shown in Figure 3b. TDFs from each cluster, selected on the basis of their putative role in plant-microbe interactions, are considered in the discussion and listed in Table 1.

**Table 1:** Selected list of melon transcripts modulated after inoculating *Fusarium oxysporum *f. sp. *melonis *(FOM) on Charentais *Fom-2 *melon plants, representative of each Cluster.

ID	Bst-Mse	Length (bp)	Melon Unigene 4.0	Uniprot	Annotation	Blast score	Category	ISPaVe1070				ISPaVe1018				ISPaVe1083			
								**2**	**4**	**8**	**21**	**2**	**4**	**8**	**21**	**2**	**4**	**8**	**21**
**CLUSTER A: TDFs specifically modulated in the incompatible interaction**								**dpi**	**dpi**	**dpi**	**dpi**	**dpi**	**dpi**	**dpi**	**dpi**	**dpi**	**dpi**	**dpi**	**dpi**

**P552**	CT-GT	193	MU45835	P48351	Catalase isozyme 3	2E-80	Defence response	0	0	1	0	0	0	0	0	0	0	0	0
**P355**	CG-CC	153	MU54667	--	putative calmodulin-related protein	3E-41	Signal transduction	0	1	1	1	0	0	0	0	0	0	0	0
**P1257**	TG-AA	119	MU54228	--	Protein translocase SEC61 complex gamma subunit	4E-40	Transport	0	-1	0	1	0	0	0	0	0	0	0	0

**CLUSTER B: TDFs specifically modulated in the compatible interaction at all time points**																			

**P431**	CC-TC	254	--	Q69LA6	Probable pyridoxal biosynthesis protein PDX1.1.	2E-28	Metabolism	0	0	0	0	1	1	-1	-2	1	1	-1	-1
**P75**	TT-TA	248	MU54623	--	Transmembrane CLPTM1 family protein	7E-56	Signal transduction	0	0	0	0	-1	-1	1	1	-1	-1	1	1

**CLUSTER C: TDFs specifically modulated in the compatible interaction, only at late time points (8 and 21 dpi)**																			

**P554**	CT-GT	210	MU45886	P09918	13S-lipoxygenase	2E-27	Defence response	0	0	0	0	0	0	0	2	0	0	0	3
**P767**	CA-CG	115	MU47701	Q39799	Endochitinase 1 precursor	2E-11	Defence response	0	0	0	0	0	0	1	2	0	0	1	2
**P1190**	CA-GT	402	MU45840	--	type I proteinase inhibitor-like protein	1E-123	Defence response	0	0	0	0	0	0	0	1	0	0	0	1
**P763**	CA-CC	167	--	Q8S0S6	Gibberellin 2-oxidase	3E-12	Metabolism	0	0	0	0	0	0	0	1	0	0	0	1
**P962**	CT-TT	175	MU48285	--	12-oxophytodienoate reductase	3E-27	Metabolism	0	0	0	0	0	0	1	2	0	0	1	2
**P257**	TT-CA	354	MU52195	Q75GK0	IAA type protein	E+00	Response to stimulus	0	0	0	0	0	0	1	2	0	0	1	2
**P1011**	TT-GA	118	--	Q9LSQ4	Indole-3-acetic acid-amido synthetase GH3.6	2E-16	Response to stimulus	0	0	0	0	0	0	0	1	0	0	0	1
**P1236**	CA-AT	53	MU51174	--	xanthine dehydrogenase-like protein	5E-19	Response to stimulus	0	0	0	0	0	0	0	2	0	0	0	2
**P1409**	TG-CA	305	--	O24174	Betaine aldehyde dehydrogenase (BADH).	2E-18	Response to stimulus	0	0	0	0	0	0	0	1	0	0	0	1
**P1430**	TA-CC	422	--	Q9S795	Betaine aldehyde dehydrogenase 1, chloroplast precursor (BADH)	4E-23	Response to stimulus	0	0	0	0	0	0	0	1	0	0	0	1
**P1418**	TG-CC	378	MU65227	B9RFG8	calmodulin-binding protein, putative, expressed	5E-113	Signal transduction	0	0	0	0	0	0	0	1	0	0	0	1
**P1479**	TC-TC	278	MU45785	P93087	Calmodulin (CaM)	3E-129	Signal transduction	0	0	0	0	0	0	0	1	0	0	0	1

**CLUSTER D: TDFs repressed or induced at different stages in both the compatible and incompatible interactions**																			

**P200**	TA-TA	445	MU48195	Q08506	ACC oxidase	E+00	Defence response	1	2	1	0	1	1	1	1	1	1	1	1
**P413**	CC-TT	214	--	B9R992	ACC oxidase 1	2E-09	Defence response	2	-1	-1	0	1	-1	-1	-2	1	-1	-1	-2
**P449**	CC-TG	155	--	Q43419	ACC oxidase.	7E-17	Defence response	1	-1	0	-1	0	-1	0	0	1	0	0	0
**P640**	CG-AC	325	--	Q40778	Allene oxide synthase	3E-24	Defence response	0	-1	1	2	-1	0	1	2	0	0	1	1
**P870**	CT-AG	407	--	Q8H1S4	ACC oxidase homolog 4	3E-26	Defence response	0	0	0	-1	0	0	0	1	0	0	0	1
**P1109**	CG-GT	206	MU47271	--	Catalase isozyme 1	2E-92	Defence response	0	0	0	-1	0	0	0	1	0	0	0	1
**P285**	TT-CC	144	MU45886	B7FDE5	13S-lipoxygenase	3E-29	Defence response	-1	-1	0	1	-1	-1	1	0	-1	-1	1	1
**P871**	CT-AG	390	--	Q9SFB5	Serine carboxypeptidase-like 27 precursor	7E-56	Defence response	-1	-1	-1	1	-1	-1	-1	-1	0	-1	-1	-1
**P1327**	TC-CG	214	MU47122	Q9SQX6	Peptidase S10, serine carboxypeptidase	5E-81	Defence response	0	1	0	0	0	0	0	1	0	0	0	1
**P534**	CT-GA	147	--	A7NSX3	Endo 1,4 beta glucanase	1E-09	Defence response	0	-1	-1	0	-1	-1	-1	-2	0	0	0	-1
**P155**	TG-GG	172	MU55410	--	aspartokinase-homoserine dehydrogenase	2E-70	Metabolism	0	1	0	1	0	0	-1	0	0	0	-1	0
**P273**	TT-CT	83	MU45201	--	HMG-CoA reductase	1E-05	Metabolism	0	0	0	-1	0	1	1	3	0	1	1	3
**P517**	CT-CG	134	--	P68173	Adenosylhomocysteinase (S-adenosyl-L-homocysteine hydrolase)	2E-13	Metabolism	0	-1	-1	-1	0	0	0	0	0	0	0	-1
**P996b**	CT-TG	151	--	Q9SP37	Adenosylhomocysteise	8E-15	Metabolism	0	-1	-1	-1	0	0	0	-1	0	0	0	-1
**P1336**	TC-GA	332	--	Q39110	Gibberellin 20 oxidase 1	2E-45	Metabolism	1	0	0	1	0	0	0	-1	0	0	0	-1
**P633**	CG-AA	190	--	B9GL14	Trehalase 1 GMTRE1	3E-19	Response to stimulus	-1	0	0	0	0	0	1	2	0	0	1	2
**P86**	TT-TT	167	MU58530	--	NDR1-like protein	2E-51	Signal transduction	1	1	0	0	0	0	1	0	0	0	1	0
**P204**	TA-TA	384	MU46993	P43187	Ca2+-binding protein	0E+00	Signal transduction	0	2	1	2	0	0	1	2	0	0	1	2
**P251**	TA-CA	64	MU52101	--	protein kinase family protein	2E-24	Signal transduction	0	1	1	1	-1	0	0	-1	1	1	-1	-1
**P873**	CT-AG	360	--	Q9ZTX8	Auxin response factor 6.	2E-19	Signal transduction	0	-1	0	1	0	0	-1	-1	0	0	-1	-1
**P1370**	TC-GC	222	MU50486	Q9FKW4	calcium-dependent protein kinase CDPK1444	1E-112	Signal transduction	0	2	1	1	0	1	1	1	1	1	1	0
**P1393**	TA-GC	247	MU51221	--	calcium-dependent calmodulin-independent protein kinase CDPK	3E-18	Signal transduction	0	1	0	0	0	1	1	1	0	1	1	1
**P1478**	TC-TC	269	--	Q9LFL1	Receptor protein kinase-like protein	2E-20	Signal transduction	0	2	1	1	0	0	1	2	0	0	1	2
**P1155**	CC-AA	108	--	Q37145	Calcium-transporting ATPase 1, chloroplast precursor	7E-10	Signal transduction	0	1	1	2	0	0	1	0	0	0	0	0
**P57**	TT-AC	237	--	Q8H0E1	AUX1-like auxin transport protein	4E-11	Transport	1	1	2	2	0	0	-1	-1	0	0	0	-1
**P1100**	CG-GA	105	MU54228	Q9SW34	Protein translocase SEC61 complex gamma subunit	2E-44	Transport	0	2	0	1	-2	-1	-1	-2	-1	-1	0	-1

This selection of representative genes modulated by infection with avirulent (ISPaVe1070 race1) or virulent (ISPaVe1018 and ISPaVe1083 race 1,2) strains of FOM is divided into clusters as defined in the results section. Identification numbers (ID) correspond to progressive numbering of bands in the gels. The primer combination used to visualize each band (Bst-Mse column) and the corresponding length of the fragment are reported, along with the accession number in the database from which the sequence was retrieved, its annotation, the corresponding BLAST score and the functional category to which each transcript was assigned. In addition, the table shows the expression profile of each transcript, in each interaction and at each time point, estimated on the basis of the band intensity with a score from -3 to 3, in comparison to the corresponding band in the uninfected controls.

The number of transcripts in each cluster indicates there is little specificity in the response to compatible and incompatible interactions, since few genes are modulated solely in one type of interaction (11 are specific to the incompatible interaction, three are specific to the compatible interaction). The data also reveal a broad response to the virulent race 1,2 strains, specific for compatibility at late time points, and that most transcripts show variable modulation over time in either of the interactions.

The clusters also show considerable differences in the pattern of transcriptional changes. In cluster C, almost all of the transcripts are induced. Only nine of the 115 transcripts are repressed, with perfect correspondence between the infection patterns of the race 1,2 strains. Cluster D includes variably expressed transcripts representing infections with either of the races. Most of the melon TDFs in this cluster increase or decrease coherently, in both compatible and incompatible interactions, with a small number of exceptions. Examples include an aspartokinase-homoserine dehydrogenase, a gibberellin oxidase, a protein translocase SEC61 complex gamma subunit and an AUX1-like protein, which are induced in the incompatible interaction and repressed in the compatible interactions. In contrast, a catalase isozyme 1, an ACC oxidase, a HMG-CoA reductase and a trehalase 1 show the opposite behavior.

### Functional categories of melon transcripts modulated by *Fusarium *infection

Each transcript was functionally annotated through a careful analysis of the scientific literature and with the aid of the Gene Ontology Database [35]http://www.geneontology.org. An overview of functional categories affected by FOM infection appears more informative when each cluster is considered separately. Because Cluster A and B contain very few genes, no diagram is provided.

Cluster A contains 11 sequences, six of which have putative annotations. Among them are transcripts corresponding to a catalase involved in the response against oxidative stress and a putative calmodulin-related protein involved in signal transduction. Cluster B contains only three sequences including a transmembrane CLPTM1 family protein, which is also induced in response to bacterial infection [36] and was identified as a possible downstream target of the heat shock regulator HsfA1a [37], and a putative pyridoxal biosynthesis protein PDX1.1, which is essential for vitamin B6 biosynthesis and has been correlated to stress tolerance and photoprotection in Arabidopsis [38].

**Figure 5:**
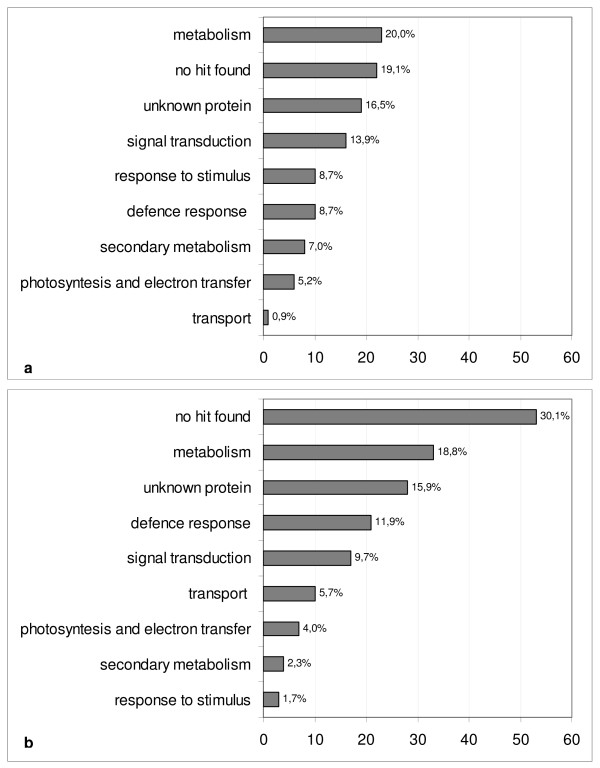
**Functional categories of melon transcripts modulated following infection with *Fusarium oxysporum *f. sp. *melonis***. Classification was carried out separately for transcripts in Cluster C (panel a) (modulated only in the compatible interaction at late time points) and in Cluster D (panel b) (modulated at different stages in both the compatible and incompatible interactions). The percentage of modulated transcripts included in each category is shown next to each bar. Data are derived from Additional File 1.

Figure 5 shows the percentages of melon genes assigned different functional categories in clusters C and D. The "Metabolism" and "Unknown protein" categories are similarly represented in both clusters (~20% and 16%, respectively). "Defense response" transcripts are also similarly represented with ~9% and 12% in clusters C and D, respectively. The "Response to stimulus" and "Secondary metabolism" categories are well represented in Cluster C, each accounting for 7-8%, while in cluster D they only represent about 2% of TDFs. The "Transport" category represents 1% of TDFs in C, but 5% in D.

### Identification of *F. oxysporum *f. sp. melonis genes expressed in melon during infection

**Figure 6:**
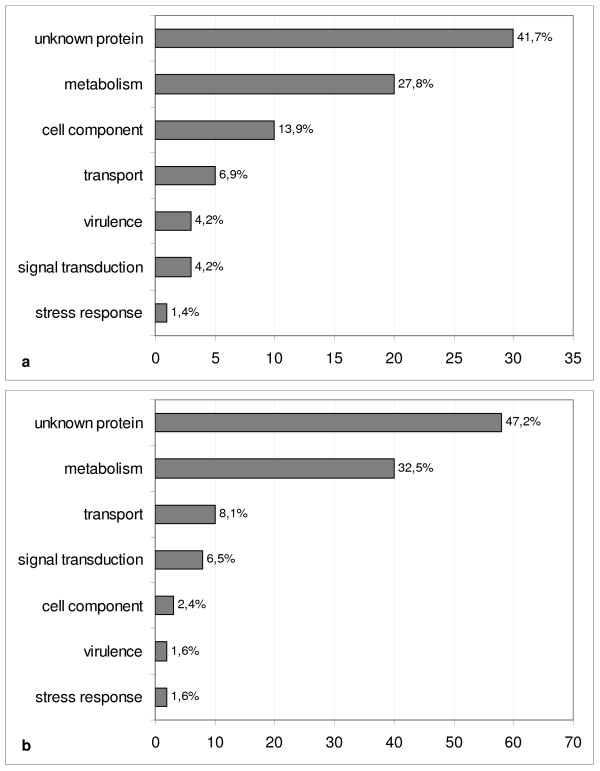
**Functional categories of fungal transcripts expressed in infected melon plants**. Classification of *Fusarium oxysporum *f. sp. *melonis *transcripts has been performed separately for those expressed only *in planta *(panel a) or both *in planta *and *in vitro *(panel b). The percentage of modulated transcripts included in each category is shown next to each bar. Data are derived from Additional File 2.

FOM genomic sequence data are scarce, so we expanded the search to include sequences from other *Fusarium *species or *F. oxysporum formae speciales *available in public databases. A total of 195 TDFs expressed *in planta *during the infection were identified as homologous to sequences assigned to *F. oxysporum *f. sp. *lycopersici*, *F. graminearum *or *F. verticilloides *(Additional File 2). Among these transcripts, 123 generated similar-sized bands in the cDNA-AFLP lanes of the fungal strains grown *in vitro*, while the remaining 72 fragments corresponded to transcripts that were not detected in fungal colonies but only *in planta *during the infection and may therefore represent factors related to virulence (Figure 6a and 6b). As expected, pathogen transcripts were detected predominantly during the late infection phase and almost exclusively in the compatible interaction, probably due to the higher fungal biomass produced in host tissues. Selected FOM transcripts detected *in planta *are listed in Table 2.

**Table 2:** Selected list of *Fusarium oxysporum *f. sp. *melonis *(FOM) transcripts expressed in melon plants during infection.

ID	Bst-Mse	Length (bp)	Accessions	Annotation	Blast score	Category	ISPaVe1070				ISPaVe1018				ISPaVe1083			
							**2 dpi**	**4 dpi**	**8 dpi**	**21 dpi**	**2 dpi**	**4 dpi**	**8 dpi**	**21 dpi**	**2 dpi**	**4 dpi**	**8 dpi**	**21 dpi**

**TDFs expressed only *in planta***																		

**P1425**	TG-CG	277	FOXT_01463	hypothetical protein similar to actin binding protein	3E-76	cell component	0	0	0	0	0	0	0	2	0	0	0	2
**P1443**	TA-CG	190	FOXG_00066	dynamin-2	6E-76	cell component	0	0	0	0	0	0	0	1	0	0	0	1
**P406**	CC-TT	479	FOXT_05654	hypothetical protein similar to 1,4-beta-glucanase	7E-124	cell wall	0	0	0	0	0	0	0	2	0	0	0	1
**P998**	CT-TG	174	FOXT_00113	chitin synthase 4	3E-52	cell wall	0	0	0	0	0	0	0	1	0	0	0	1
**P1261**	TG-AT	209	AF080485	Fusarium oxysporum f. sp. lycopersici pectate lyase	2E-87	cell wall-degrading	0	0	0	0	0	0	0	1	0	0	0	2
**P553**	CT-GT	235	FOXG_09638	endo-1,4-beta-xylanase 2 precursor	7E-101	cell wall-degrading	0	0	0	1	0	0	0	0	0	0	0	0
**P1362**	TC-GC	377	FOXT_13415	endo-1,4-beta-xylanase	9E-145	cell wall-degrading	0	0	0	0	0	0	0	2	0	0	0	1
**P32**	TT-AA	148	FOXG_06061	squalene synthetase	1E-42	metabolism	0	0	0	0	0	0	0	1	0	0	0	1
**P675**	CG-TT	259	FOXT_08412	protein similar to phosphoserine phosphatase	1E-114	signal transduction	0	0	0	0	0	0	0	1	0	0	0	1
**P1473**	TG-AG	224	FOXT_00565	hypothetical protein similar to calnexin	4E-102	signal transduction	0	0	0	0	0	0	0	1	0	0	0	1
**P842**	CT-AA	382	FOXG_03102	hypothetical protein similar to MADS box protein	4E-131	signal transduction	0	0	1	1	0	1	1	2	0	1	1	2
**P1331**	TC-CG	165	FVET_00351	hypothetical protein similar to vps28 protein	3E-24	transport	0	1	0	0	0	0	0	1	0	0	0	1
**P888**	TC-TA	449	FOXT_11289	plasma membrane ATPase	1E-178	transport	0	0	0	0	0	0	0	1	0	0	0	1
**P1014**	TT-GA	140	FOXT_12267	hypothetical protein similar to hexose transporter	8E-25	transport	0	0	0	0	0	0	0	2	0	0	0	1
**P1066**	CC-GC	380	FOXT_11113	mitochondrial phosphate carrier protein	1E-177	transport	0	0	0	0	0	0	0	1	0	0	0	1
**P1232**	CA-AT	341	FOXT_10521	mitochondrial import inner membrane translocase	1E-63	transport	0	0	0	0	0	0	0	1	0	0	0	1
**P819**	TC-AC	359	FOXG_09125	hypothetical protein similar to avenacinase	6E-117	virulence	0	0	0	0	0	0	0	3	0	0	0	1
**P1258**	TG-AT	468	FOXG_09782	siderophore iron transporter mirB	0	virulence	0	0	0	0	0	0	0	1	0	0	0	1
**P1482**	TC-TC	187	FOXT_03354	hypothetical protein similar to Fum16p	3E-81	virulence	0	0	0	0	0	0	0	2	0	0	0	2

**TDFs expressed both *in planta *and *in vitro***																		

**P417**	CC-TT	168	FOXT_11103	hypothetical protein similar to sterol esterase precursor	6E-57	lipid metabolism	0	0	0	0	0	0	0	2	0	0	0	1
**P292**	TT-CG	180	FOXT_00536	UTP-glucose-1-phosphate uridylyltransferase	1E-77	metabolism	0	0	0	0	0	0	0	2	0	0	2	2
**P305**	CG-CA	199	FOXG_09447	pyruvate dehydrogenase E1 component subunit alpha	2E-88	metabolism	0	0	0	0	0	0	0	2	0	0	0	1
**P344**	CG-CC	363	FOXG_11520	NAD-specific glutamate dehydrogenase	1E-103	metabolism	0	0	0	0	0	0	0	3	0	0	0	2
**P1211**	CA-GC	159	FOXG_10419	malate dehydrogenase, mitochondrial precursor	1,00E-67	metabolism	0	0	0	0	0	0	0	2	0	0	0	2
**P1333**	TC-CG	140	FOXT_11398	UDP-glucose 6-dehydrogenase	4,00E-58	metabolism	0	0	0	0	0	0	0	3	0	0	0	3
**P1486**	CC-CA	226	FOXT_07937	glutathione reductase	1E-89	metabolism	0	0	0	0	0	0	0	1	0	0	0	1
**P1280**	TG-TC	358	FOXT_00266	hypothetical protein similar to multiubiquitin	1E-60	protein metabolism	0	0	0	0	0	0	0	1	0	0	0	1
**P201**	TA-TA	443	FOXG_03710	hypothetical protein similar to proteasome regulatory	0	protein metabolism	0	0	0	0	0	0	0	1	0	0	0	1
**P1398**	TA-GG	286	FOXT_00231	proteasome component C5	2E-139	protein metabolism	0	0	0	0	0	0	0	1	0	0	0	1
**P304**	CG-CA	215	FOXT_10713	hypothetical protein similar to Ca2+ ATPase	4E-68	signal transduction	0	0	0	0	0	0	0	1	0	0	0	1
**P960**	CT-TT	187	FOXG_05517	cAMP-dependent protein kinase regulatory subunit	6E-79	signal transduction	0	0	0	0	0	0	0	1	0	0	0	1
**P469**	CT-CA	294	FOXT_12260	peroxidase/catalase 2	7E-148	stress response	0	0	0	0	0	0	0	2	0	0	0	2
**P1134**	CG-GG	352	FOXT_01866	sorting nexin-41	0	transport	0	0	0	0	0	0	0	1	0	0	0	1
**P464**	CC-TG	136	FOXT_09879	hypothetical protein similar to neutral amino acid permease	8E-40	transport	0	0	0	0	0	0	0	2	0	0	0	1
**P611**	CT-GC	284	FOXG_09522	hypothetical protein similar to metalloreductase	1E-139	transport	0	0	0	0	0	0	0	3	0	0	0	3
**P1293**	TC-CA	396	FVET_08954	hypothetical protein similar to vacuolar sorting protein 35	0	transport	0	0	0	0	0	0	0	1	0	0	0	1
**P365**	CG-CG	426	FOXT_12915	arginase	0	virulence	0	0	0	0	0	0	0	1	0	0	0	1
**P610**	CT-GC	291	FOXT_05960	hypothetical protein similar to peroxisomal PEX11	1E-136	virulence	0	0	0	0	0	0	0	2	0	0	0	1

Selected FOM transcripts identified in infected melon plants, either specifically *in planta*, or both *in planta *and *in vitro*. Identification numbers (ID) correspond to progressive numbering of bands in the gels. The primer combination used to visualize each band (Bst-Mse column) and the corresponding length of the fragment are reported, along with the accession number in the database from which the sequence was retrieved, its annotation, the corresponding blast score and the functional category to which each transcript was assigned. In addition, the table reports the expression profile of each transcript, in each interaction and at each time point, estimated according to band intensity with a score from 1 to 3.

Fungal genes expressed only *in planta *(Figure 6a) or *in planta *and *in vitro *(Figure 6b) were also assigned functional categories based on careful literature evaluation. This allowed us to identify some interesting differences, namely in the "Cell component" and in the "Virulence" categories, which are represented more *in planta *than *in vitro*. Other categories show similar percentages in both groups.

### Detection of fungal transcripts differentially expressed among strains grown *in vitro*

We identified 199 bands that were differentially expressed among the three FOM strains grown *in vitro*, 75 of which were expressed uniquely *in vitro *and were selected for amplification and sequencing. For the remaining 123 TDFs, similar sized bands were also present *in planta *and in most cases the corresponding cDNAs had already been excised from the infected melon lanes (see above). Of the 75 TDFs expressed only *in vitro*, 53 were specifically expressed by strain ISPaVe1070 (race 1), and 22 (11 each) were specifically expressed by the two strains of race 1,2 (Additional File 4). Searching the *Fusarium *database [39] revealed sequences similar to at least one *Fusarium *gene for 46 fragments, 15 of which were annotated. Another 29 sequences did not match any public sequences and could represent novel *F. oxysporum *genes with a putative role in virulence.

### Validation of representative genes by real-time RT-PCR

The expression profiles of seven modulated melon transcripts were analyzed by real-time RT-PCR to validate cDNA-AFLP data (Table 3). Genes were chosen among those expressed *in planta *as representative of the different clusters, with a preference for defense-related genes. The RT-PCR expression profiles of six genes agreed strongly with the cDNA-AFLP data, whereas the protein translocase SEC61 complex gamma subunit appeared to be induced to a significantly greater extent according to the RT-PCR results. The identity and expression of five fungal TDFs expressed *in planta *was confirmed using the same technique (Table 4). Minor discrepancies are observed when expression levels are measured using different techniques [40,41] but the RT-PCR and cDNA-AFLP data are largely concordant, confirming the reliability of the results.

**Table 3:** Real time RT-PCR expression analysis of selected melon genes.

ID	Homology	E-value	Annotation	ISPaVe strain/dpi	I/R	Fold-change (Infect. vs. mock)	SD
**P200**	MU48195	0	ACC oxidase	1070/4 dpi	I	7.04	2.80
**P557**	MU51460	3E-45	Respiratory burst oxidase homolog protein C	1070/4 dpi	I	61.92	14.73
**P1100**	MU54228	2E-44	Protein translocase SEC61	1018/2 dpi	R	1.87	0.37
**P1370**	MU50486	1E-112	calcium-dependent protein kinase CDPK1444	1070/4 dpi	I	3.81	0.66
**P554**	MU45886	2E-27	13S-lipoxygenase	1018/21 dpi	I	1.26	0.14
**P767**	MU47701	4E-50	Endochitinase 1 precursor	1018/21 dpi	I	473.05	146.45
**P769**	MU43951	6E-61	UBQ14 (ubiquitin 14)	1018/21 dpi	I	25.34	24.29

Seven melon transcripts, differentially expressed following *Fusarium oxysporum *f. sp. *melonis *(FOM) infection according to cDNA-AFLP experiments, were selected for validation by real time RT-PCR. Each gene was validated at the same time point and with the same FOM strain that generated a differential cDNA-AFLP profile. The FOM strains are ISPaVe170 race1 and ISPaVe1018 race 1,2. Results are shown as fold change values in infected samples in comparison to mock-inoculated controls and are the average of three technical replicates. I/R: induced or repressed according to cDNA-AFLP experiments. SD: standard deviation.

**Table 4:** Real time RT-PCR expression analysis of selected *Fusarium oxysporum *f. sp. *melonis *(FOM) genes.

ID	Homology	E-value	Annotation	ΔCt	SD
**P493**	FOXT_12671	5E-152	Fusarium oxysporum f.sp lycopersici aspartate aminotransferase	3.43	0.11
**P135**	FOXT_08523	1E-48	Fusarium oxysporum f.sp lycopersici acyl-CoA desaturase	16.11	0.41
**P1315**	FOXG_05190	2E-79	Fusarium oxysporum f.sp lycopersici E3 ubiquitin-protein ligase	3.99	0.31
**P819**	FOXG_09125	2E-117	Fusarium oxysporum f.sp lycopersici hypothetical protein similar to avenacinase	10.24	0.04
**P1403**	FOXT_13204	7E-51	Fusarium oxysporum f.sp lycopersici predicted protein	7.06	0.77

Five *Fusarium oxysporum *f. sp. *melonis *transcripts, expressed *in planta *during infection according to cDNA-AFLP experiments, were selected for validation by Real time RT-PCR. Data are shown as the difference between the Ct of the gene analyzed and the Ct of melon actin (AY859055), used as a normalizer.

## Discussion

Melon is an important horticultural crop and is rapidly becoming a popular research model for cucurbits, thanks to the increasing availability of genomic resources. Several diseases affect melon production, but molecular investigations of plant-pathogen interactions in melon are still rare [42]. Recently, a melon array resource has been developed, allowing the transcriptomic analysis of several physiological and pathological conditions [43]. However, the melon genome has not been fully sequenced, so alternative transcriptomic approaches allowing novel gene discovery are still important. The cDNA-AFLP method is particularly appropriate for gene expression studies in non-model species and it also allows identification of both plant and pathogen genes expressed during infection, an important pre-requisite for the identification of pathogenicity and virulence factors and thus for the identification of targeted control strategies [44-48,20]. Our study provides the first large-scale investigation of gene expression changes that occur when melon is infected with FOM, the most important melon pathogen, and is the first to compare compatible and incompatible interactions in the same genetic background (Charentais *Fom-2*).

### Analysis of *F. oxysporum *f. sp. melonis colonization in melon stems

Because few researchers have investigated FOM infections in melon, the site and timing of recognition is currently unknown, which makes difficult to propose suitable time points for molecular analysis. We therefore began this investigation by characterizing the infection process in melon plants inoculated with avirulent FOM race 1 and virulent race 1,2. Disease progression was monitored using the same approach (plating stem fragments at different time points) that has been successful in tomato [49]. Colonization followed a similar trend to that reported for *F. oxysporum *f. sp. *lycopersici *in tomato [49], i.e. the fungus distribution was discontinuous in all combinations from 2-8 dpi, then continuous from 14-21 dpi with distinct patterns in the incompatible and compatible combinations. From 14 dpi onwards, symptoms became obvious in the compatible interaction as generally reported in the literature [49,11,20]. Whereas the two virulent strains fully colonize the stem, colonization by the avirulent strain is reduced, and at 18 and 21 dpi the height reached in stems is significantly lower than that reached at 2 and 4 dpi. These findings suggest that the plant may attack the invading pathogen and reduce its vitality. The data were confirmed by real-time PCR, indicating a progressive reduction in the amount of fungus present at later time points in the incompatible interaction (data not shown). Di Pietro and colleagues [2] found that, having reached the xylem, the fungus remains exclusively within the vessels using them to colonize the host rapidly, mainly through the production of microconidia rather than mycelia which, in turn, progressively grows inside the xylem inducing vessel clogging. In contrast to this prominent microconidia model [20,23], studies using GFP-labeled *F. oxysporum *have shown that neither conidiophores nor microconidia are found in Arabidopsis or tomato xylem [21,22]. The response to infection may be affected by inoculum concentration, the age of the plant, the duration of exposure to the inoculum, and the type of substrate for plant growth (e. g. sand or soil) [11,27]. The assessment time points may also play an important role in the picture that emerges of the host/pathogen genetic responses. Nevertheless, differences in the infection process are likely to occur among different *formae speciales *and between different experimental designs.

### Genetic elements of host colonization and pathogenicity

Most transcriptomics studies involving *F. oxysporum *have focused on the interactions that occur in the xylem, and these studies suggest that the main resistance responses occur within or along the vessels. In this context, genes that are expressed solely *in planta *and not in artificial culture are the most interesting because they are likely virulence factors (Table 2 and Additional File 2). We identified 195 genes that were expressed *in planta*, 72 of which (~37%) were not expressed under artificial culture conditions and therefore represent putative virulence factors. Interestingly, only 11 out of 218 genes in cotton plants infected with *F. oxysporum *f. sp. *vasinfectum *were expressed specifically *in planta *[50]. The group of putative virulence factors identified in our analysis included plant cell wall degrading enzymes (CWDEs), represented by five transcripts encoding pectate lyases, endo-1,4 beta xylanases and endo-1,4 beta glucanases, possibly activated by interaction with the host. Among these transcripts, an endo-1,4-beta-xylanase 2 precursor is the only sequence peculiar to race 1, induced in the incompatible interaction, while the other four TDFs are specific to the race 1,2 strains. Like most fungi, *F. oxysporum *secretes CWDEs during either penetration or colonization [23]. Although the inactivation of individual CWDE- or protease-encoding genes might not have a detectable impact on virulence (reviewed in [2]), possibly because of functional redundancy, their activity is crucial in the process of fungal colonization. Active fungal growth is also documented by the specific *in planta *expression of several genes related to carbohydrate and lipid metabolism, among them a squalene synthase involved in sterol biosynthesis. Sterols facilitate normal membrane function by controlling their fluidity, but they have also been implicated as ligands for nuclear receptors directly affecting transcription and signal transduction pathways [51]. Other examples include genes for cytoskeleton components (hypothetical protein similar to actin binding protein and dynamin-2) and a chitin synthase gene. Class V chitin synthase is a pathogenicity determinant in *F. oxysporum *and a mediator of protection against plant defense compounds [26].

Three other *in planta*-specific TDFs seem particularly important in terms of virulence. These represent genes encoding homologs of an avenacinase, a fumonisin 16p, and a siderophore iron transporter (*mir*B). There is increasing evidence that mycotoxin production may enhance pathogen virulence, especially fumonisins and some trichothecenes [52]. Fumonisin enhances the ability of *F. graminearum *to cause wheat head blight, one of the most important wheat diseases in the world [53]. It has been reported that mycotoxin production can be induced in fungi following the perception of the oxidative burst produced by the plant in response to infection, and could enhance pathogenicity by reducing the oxidative status of the fungal cell. Interestingly, the gene encoding the fungal toxin fumonisin was strongly and specifically expressed *in planta *only by virulent strains.

Avenacina is a hydrolytic enzyme that can degrade the oat saponin avenacine, and was first recognized as an essential pathogenicity factor in the take-all fungus *Gaeumannomyces graminis *var. *avenae*. Saponins, glycosides with soap-like properties that disrupt membranes, are a class of phytoanticipins. The role of saponin detoxification remains controversial in other plant-pathogen interactions [54,55]. However, the saponin-degrading tomatinase from *F. oxysporum *f. sp. *lycopersici *has recently been confirmed as a virulence factor in tomato, by targeted disruption and over-expression of the corresponding gene [56]. In melon, we found that the avenacinase transcript is not only expressed specifically *in planta*, but is also differentially expressed between the two 1,2 strains, with higher levels produced by ISPaVe1018. To our knowledge, this is the first evidence to support a role for saponin-detoxifying enzymes in FOM infection. The siderophore iron transporter *mir*B gene may also represent a virulence factor because siderophores (low-molecular-mass iron chelators that facilitate iron uptake and storage) are crucial for fungal pathogenicity in both animals and plants, and also maintain plant-fungal symbioses [57]. The final group of FOM genes expressed only *in planta *includes several involved in transport and intracellular trafficking, and three related to signal transduction, with similarity to a calnexin involved in calcium-regulated protein folding [58], a phosphoserine phosphatase and a MADS box protein.

Although expressed both *in planta *and *in vitro*, a peroxisomal biogenesis factor PEX11 and an arginase coding gene are also worth mentioning (Table 2). Peroxisomes are single-membrane-bound organelles which, in filamentous fungi, are involved in the β-oxidation of fatty acids, peroxide detoxification and the occlusion of septal pores [27]. Peroxisomal function and fatty acid metabolism are required for fungal virulence. In *F. oxysporum*, four different *Pex *genes (PEX1, PEX10, PEX12 and PEX26) were identified as potential pathogenicity genes in a recent insertional mutagenesis screen, and the requirement for full pathogenicity was verified for two of them (PEX12 and PEX26) by complementation with the intact genes [59]. Arginase regulates the production of nitric oxide (NO), which is induced in a jasmonate-dependent manner in response to wounding and is strongly implicated in the activation of disease resistance genes [60,31]. In microorganisms, arginase activity has been correlated with pathogenicity [61] and was shown to act as a bacterial survival mechanism by downregulating host nitric oxide production [62]. Other transcripts expressed by FOM *in planta*, specifically or otherwise, are involved in ubiquitinylation and protein degradation, both of which are necessary for pathogenicity in *F. oxysporum *f. sp. *lycopersici *[27], and in different aspects of fungal metabolism.

### Differentially expressed genes among *F. oxysporum *f. sp. melonis strains *in vitro*

One major problem in FOM diagnosis is the identification of isolates at the race level. In melon-growing areas, the introduction of races that can overcome resistance in cultivated genotypes may result in dramatic crop losses. At present, no molecular tools are available to replace the time-consuming race-determination tests. We identified a number of transcripts with differential expression profiles between the two races (Additional File 4). Although differences in gene expression cannot be used directly as genetic markers of race identity, TDFs could be used as 'fingerprints' for this purpose. In addition, the differential virulence of the two 1,2 strains demonstrated by the host colonization pattern, could also be fingerprinted using TDFs that are differentially expressed between ISPaVe1018 and ISPaVe1083. Unfortunately, most TDFs in this category either matched hypothetical protein sequences in public databases or did not generate hits at all, and therefore do not allow speculation about the possible metabolic differences between the two races or between the two strains of FOM race 1,2.

### Large-scale transcriptional changes underlie disease development

Transcriptional changes associated with resistance responses occur within the first 2 dpi, and are maintained with few changes thereafter (Figure 4). However, only 11 melon transcripts (Cluster A) are specific for the incompatible interaction. The largest group of modulated genes (Cluster D) is expressed in a non-specific manner, with variable modulation throughout the experiment, in both the incompatible and compatible interactions. The establishment of compatibility is characterized by a slightly delayed but progressive increase of the number of genes involved, underlying the significant metabolic disturbances that might be associated with symptom development. The majority of these changes are included in Cluster D and are thus non-specific up to 8 dpi, but are followed by a sudden wave of susceptibility-specific transcriptional changes at 21 dpi, almost completely conserved between the virulent strains ISPaVe1018 and ISPaVe1083.

Although the precocity of the resistance response is expected, the small number of genes involved is unexpected. Incompatible interactions commonly involve large-scale transcriptional reprogramming toward defense, which is generally more intense and rapid than in corresponding compatible interactions [63,64,28]. However, vascular diseases may represent a peculiar situation, in which symptom development and consequent damage could depend not only on the pathogenetic activity of the fungus but also the strength and timing of the host response. This was indicated by pioneering research in which delayed formation of tyloses in susceptible genotypes eventually contributes to vessel clogging [23]. In agreement with the above, our data suggest that more striking changes in gene expression accompany disease and symptom development than resistance, thus resistance might depend more on the ability to tolerate the infection, avoiding reactions.

### Transcriptional changes in the compatible interaction

Although cluster analysis and functional annotation of the identified melon transcripts provided an overview of the transcriptional changes occurring in infected melon plants, the limited availability of sequence data made it difficult to draw firm conclusions about the molecular events occurring in infected plants. Of the 115 TDFs identified in Cluster C, 41 did not match any database sequences or matched sequences that have yet to be annotated. The remaining 74 TDFs encode well-known components of disease resistance responses and related signal transduction cascades, such as calmodulin and calmodulin-binding proteins, transcription factors, a 12-oxophytodienoate reductase, and a 13S-lipoxygenase involved in jasmonic acid biosynthesis [65], and enzymes involved in the biosynthesis of secondary metabolites acting as antimicrobial compounds, or in a general stress responses, such as xanthine dehydrogenase and betaine aldehyde dehydrogenase [66,67]. Genes encoding pathogenesis-related proteins such as endochitinase, beta-1,3 glucanase and a type I proteinase inhibitor-like protein were also specifically modulated in the compatible interaction. Altogether, transcripts related to the defense, response to stimulus and secondary metabolism categories accounted for ~25% of modulated TDFs in Cluster C. These findings further support the hypothesis that a delayed defense response might indeed be responsible for symptom development.

Cluster C also contained genes potentially involved in the establishment of susceptibility, such as those related to auxin accumulation. Several reports indicate that an increase in auxin levels in the cell can contribute to disease susceptibility [68] and that a similar increase can be induced by pathogens in order to facilitate colonization. TDFs with homology to an indole-3-acetic acid-amino synthetase and to an IAA-type protein Q75GK0 [68] are specifically induced in the compatible interaction. However, other genes in Cluster D that induce auxin signaling (e.g. auxin response factor 6 and AUX1-like auxin transport protein) are repressed by both virulent strains, but induced by the avirulent strain at 21 dpi. The overall picture is therefore complex and suggests that the compatible interaction mainly involves transcriptional changes that are otherwise typical of effective resistance responses. It is tempting to speculate that the recessive resistance identified in Asian accessions might be related to the lack of a plant reaction and thus to better tolerance of the infection process [20].

### Transcriptional changes in the incompatible interaction

Resistance responses are generally characterized by rapid and extensive reprogramming of transcriptional activity, especially in race-specific interactions. However, that resistance of Charentais *Fom-2 *to FOM race 1, although mediated by a single R-type resistance gene, is not complete, since the fungus can always be reisolated from the stem of Charentais *Fom-2 *plants. In our model system we noted surprisingly few transcriptional changes specifically associated with the incompatible interaction (11 TDFs, Cluster A). These included a calmodulin-related protein, stably upregulated from 4 dpi onwards, which plays an important role in the transduction of calcium signaling and could be involved in the resistant response (as already demonstrated in other species [69]) and a catalase that may protect the pathogen from the strong oxidative burst associated with resistance.

Additional information about the resistance response could be found by analyzing genes that are modulated in both interactions (Cluster D) but with a peculiar pattern (e.g. earlier or more strongly in the incompatible interaction), or those showing a reverse modulation of gene expression in one or the other interactions (which might reveal specific requirement in each interaction). An interesting finding is that many of the genes modulated earlier or more strongly during resistance are classified as signal transduction-related genes (Additional File 1). These include several transcripts involved in calcium signaling (calcium-transporting ATPase, calcium dependent protein kinase and calcium-binding protein), transcription factors, kinases and a homolog of NDR1 (non race-specific disease resistance 1). This last gene is induced at 2 and 4 dpi following infection with race 1 and only at 8 dpi following infection with race 1,2. NDR1 was originally identified in Arabidopsis as a factor required for resistance to both bacterial and fungal pathogens [70] and it is known to mediate resistance controlled by R genes of the nucleotide binding site leucine rich repeats (NBS-LRR) class, which is distinct from the Toll/interleukin receptor (TIR) class [71]. *Fom-2 *in Charentais *Fom-2 *plants is indeed a non-TIR R gene, although with a peculiar structure that lacks the typical N-terminal coiled-coil domain [72]. Therefore, it seems plausible that its action might require NDR1.

Other genes modulated earlier in the establishment of resistance include two adenosylhomocysteinases, an aspartokinase-homoserine dehydrogenase and a serine carboxypeptidase. An Arabidopsis adenosylhomocysteinase encoded by the gene HOG1 (homology dependent gene silencing 1) is required for DNA-methylation gene silencing [73]. The involvement of RNA-silencing machinery in plant innate immunity has recently been demonstrated not only against viruses but also bacterial and fungal pathogens, including *Verticillium *in Arabidopsis [74,75]. The same transcript increases in aphid-infested sorghum plants [76]. The potential involvement of these candidate genes in Charentais *Fom-2*- controlled resistance could be the object of future investigations.

Most genes in Cluster D are not differentially modulated in the incompatible and compatible interactions. Interesting examples include TDFs related to ACC oxidases. These enzymes participate in the last step of ethylene biosynthesis and are involved in the response to stress and to pathogens, but are also implicated in senescence, necrosis and disease development. Ethylene has been associated with both wilting and resistance against vascular diseases [77]. We detected four transcripts with similarity to ACC oxidases. These showed variable expression profiles, but there was no difference between compatible and incompatible interactions, which suggests that ethylene might be involved in both susceptibility and resistance. In melon, different ACC oxidase genes are induced differentially during development and pathogen infection [78]. The same variable modulation has been detected for other transcripts possibly related to jasmonate biosynthesis, such as allene oxide synthase and the 13S-lipoxygenase mentioned above.

## Conclusions

In conclusion, our data suggest that resistance against FOM in melon involves only limited transcriptional changes, and that wilting symptoms could derive, at least partially, from an active plant response.

A small but important collection of FOM transcripts were shown to be expressed specifically *in planta*, and not in the same fungal strains growing *in vitro*, providing excellent candidate virulence factors which can be investigated further to learn more about the molecular basis of host-pathogen interactions in melon. Finally, race-specific genes were expressed in fungal colonies *in vitro *as well as *in planta*, suggesting they could be developed as markers in molecular race-determination assays that could replace the current laborious inoculation-based methods.

## Methods

### Plant material

Seeds from melon (*Cucumis melo *L.) genotype Charentais *Fom*-*2 *(resistant to races 0 and 1, and susceptible to race 1,2) were surface sterilized with 1% NaOCl for 20 min and incubated in sterile distilled water at 4°C overnight. The seeds were pre-germinated on filter paper, and seedlings were cultivated in plastic pots filled with sterilized soil in the greenhouse at 25 ± 2°C with 80-90% relative humidity.

### Pathogen material and production of the inoculum

Virulent *F. oxysporum *f. sp. *melonis *Snyder & Hans. (FOM) strains were obtained from the fungal collection of the Plant Pathology Research Center (CRA-PAV) [79]. Three strains were used as inoculum, namely ISPaVe1070 (race 1), and ISPaVe1018 and ISPaVe1083 (race 1,2). Race designation had been achieved by inoculation on different hosts according to the nomenclature proposed by Risser *et al*. [5]. Inoculums were produced by growing each strain on 90-mm Petri dishes containing potato-dextrose agar (PDA, Oxoid). Fourteen-day-old cultures grown at 24°C were flooded with sterile distilled water and gently scraped with a sterile glass rod to obtain a spore suspension. This was filtered through two layers of cheesecloth and the filtrate was diluted to obtain the inoculum at a concentration of 1 × 10^6 ^conidia/ml.

### Inoculation procedure

Charentais *Fom-2 *melon seedlings were inoculated at the four-to-five true leaf stage [18]. The roots of each seedling were gently washed in tap water, pruned by approximately 1 cm and dipped for 30 min in the conidial suspension. Control seedlings were dipped in sterile distilled water. Seedlings were then transferred into plastic pots filled with sterilized soil and maintained in the greenhouse at 25 ± 2°C with 80-90% relative humidity. For each fungal strain, a total of 72 plants (eight reisolation times after inoculation multiplied by nine replicates) was used to investigate vascular colonization, and 20 plants (four plants per each time point) were used for RNA extraction and transcriptomic analysis.

### Vascular colonization

After inoculation, seedlings were monitored for fungal colonization along the stem by reisolation. The experiment was concluded at 21 dpi, when all plants undergoing the compatible interaction (Charentais *Fom-2 *inoculated with race 1,2) displayed obvious and severe wilting symptoms. Nine plants for each strain were cut at the stem base 1, 2, 4, 8, 14, 16, 18 and 21 dpi, and were defoliated. After discarding the basal 15 mm, the stems were cut into 12 sections, each 5 mm in length, to a maximum height of 75 mm. Sections were placed on PDA, incubated at room temperature for 8 days and examined every day for the appearance of fungal outgrowths. A completely randomized distribution was adopted for the melon plants kept in the greenhouse as well as for the Petri-dishes with stem sections incubated in the laboratory.

### Data analysis

Vascular colonization was scored according to the frequency of successful reisolation in stem sections arranged in four height classes measured from the stem base: 15-30 mm, 30-45 mm, 45-60 mm and 60-75 mm. Percentage values grouped in the four height classes were subjected to a two-way ANOVA for each height class and for the total of the four classes. The two factors considered were strain and time (dpi). The data did not match the parametric ANOVA requirements (normal data distribution and homogeneous variance) with any transformation, so the non-parametric Monte Carlo permutation test was used instead. The probabilities of the main effects of each factor were generated by restricting permutations within the levels of the other factor [80], whereas the interaction between strain and time was tested by unrestricted permutations after the calculation of residuals [81]. The statistical test used for the main factors was the sum of squares between groups (Q_b_), whereas the test used for interaction was the pseudo F-ratio (F = Q_b_/Q_w_, where Q_w _is the sum of squares within groups). Because of interactions between factors present in all five two-way ANOVA tests, the effect of time was tested separately for each strain in a one-way ANOVA either for each height class or for the total of the four classes. Follow-up tests of the differences between times were performed by considering all possible pairwise contrasts. In this case, to avoid inflation of the type I error rate, a Bonferroni-corrected significance level of P ≤ 0.0018 (P < 0.05 divided by 28, which is the total number of possible contrasts between the eight reisolation times) was calculated and used as minimum nominal P-value to obtain an actual P ≤ 0.05 value. The statistical results refer to the analysis performed on the total of the four height classes for each strain.

To characterize the continuity of the distribution of the fungus along the stem, a continuity index was calculated based on the reisolation data. The index was determined for each plant by considering the presence or absence of the fungus in the pairs of subsequent stem sections and assigning a value of 1 when the fungus was reisolated or not reisolated in both sections and a value of 0 when it was reisolated only in one of the two sections. The index was then calculated by averaging the obtained values.

### RNA extraction procedure

For each plant tissue sample, ~2 g of stem segments (four plants) were excised with a sterile razor blade, dehydrated in liquid nitrogen and stored at -80°C. Total RNA was extracted using TRIzol reagent (Invitrogen) and treated with DNase (Sigma-Aldrich) following the manufacturer's instructions. For fungal colonies, total RNA was extracted from frozen single-spore colonies (60-150 mg), grown for 8 days on PDA at 24°C, with the RNeasy Mini kit (Qiagen) following the manufacturer's protocol for plant tissues.

### cDNA-AFLP analysis

We used the cDNA-AFLP protocol described by Vos *et al*. [82] and Bachem *et al*. [83] with the modifications and primers described by Breyne *et al*. [84] which permit the visualization of a single cDNA fragment for each mRNA present in the original sample, reducing the output sequence redundancy. Double-stranded cDNA was synthesized from 2.5 μg total RNA using the Superscript II reverse transcription kit (Invitrogen) and a biotinylated oligo-dT primer (Promega).

After pre-amplification, the mixture was diluted 600-fold and 5 μl was used for selective amplification with 128 primer combinations, carried out with one selective nucleotide added on the ^33^P-labeled *Bst*YI primer and two selective nucleotides on the *Mse*I primer. We used the following touch-down PCR conditions: 2 min denaturation at 94°C followed by 30 s denaturation at 94°C, 30 s annealing at 65°C, 60 s extension at 72°C for 13 cycles (scaledown of 0.7°C per cycle); 30 s denaturation at 94°C, 30 s annealing at 56°C, 60 s extension at 72°C for 23 cycles, and 5 min at 72°C. Selective [γ-^33^P]ATP-labeled amplification products were separated on a 6% polyacrylamide gel in a Sequi-Gen GT Sequencing Cell (38 × 50 cm) (Bio-Rad) running for 2.5 h at 115 W and 50°C. Gels were dried onto 3 MM Whatman paper for 2 h at 80°C on a Gel Dryer Model 583 (Bio-Rad) and marked with Glogos II Autorad Markers (Stratagene) before exposing to Kodak Biomax MR films, for 12-16 h.

### Sequence analysis of cDNA-AFLP fragments

Bands corresponding to differentially expressed genes were excised from the gels with a surgical blade and the eluted DNA was reamplified using non-labeled primers identical to those employed for selective amplification and the following PCR conditions: 15 min denaturation at 94°C, 40 s denaturation at 94°C, 60 s annealing at 56°C, 40 s extension at 72°C for 35 cycles, and 5 min at 72°C. The quantity of each reamplified bands were checked on a 1.8% agarose gel against the 1650-bp fragment of the DNA ladder 1 Kb plus (Invitrogen). PCR products were purified with MultiScreen PCR μ96 plates (Millipore) and sequenced directly (BMR Genomics). Sequence information was obtained by comparing nucleotide and protein sequences in the available public databases by BLAST sequence alignment [85]. Homology searching was carried out against the following databases: NCBI [34], Cucurbit Genomic Database Melon Unigene ver. 4.0 [32], UNIPROT database [33] and Fusarium Comparative Database [39]. Sequences were manually assigned to functional categories based on the analysis of scientific literature and also with the aid of the information reported for each sequences by the Gene Ontology Consortium [35], where available.

Sequence data from this article have been deposited in GenBank, Accession Numbers: HO867279- HO867981.

### Real-time RT-PCR analysis

Real-time RT-PCR was carried out on pools of RNA derived from two independent biological experiments. All samples were examined as three technical replicates. Samples were prepared from whole stems of infected and mock-inoculated plants (T0) corresponding, for each gene tested, to the same interaction type and time point that indicated differential expression in the equivalent cDNA-AFLP experiment. The FOM strains were ISPaVe170 (race1) and ISPaVe1018 (race 1,2 w). Total RNA was treated with RNase-free DNase (Sigma-Aldrich) according to the manufacturer's instructions, and 3 μg was then used for reverse transcription on Ready-To-Go you-prime first-strand beads (GE Healthcare). Then 5 μl of 1:10 diluted cDNA samples was used as the qRT PCR template in a 25-μl total volume containing 0.4 μM gene-specific primers and 12.5 μl platinum SYBR Green qPCR SuperMix with ROX (Invitrogen). All samples were examined in three technical replicates. Experiments were carried out in a Mx3000P QPCR Systems (Stratagene) with the following thermal cycling profile: 95°C for 10 min; 40 cycles of 95°C for 30 s, 55°C for 30 s, 72°C for 30 s. Each real-time assay was tested in a dissociation protocol to ensure that each amplicon was a single product. Relative quantification of gene expression was performed using the housekeeping gene actin [31]. The actual stability of actin expression was tested in preliminary experiments, calculating the coefficient of variation (CV = 0.047) of the threshold cycle for actin amplification in all infection conditions and in mock inoculated controls [86]. The primers were designed according to the melon actin sequence in GenBank (AY859055): forward, 5'-CCC TGG TAT TGC AGA CAG GA-3' and reverse, 5'-ACA TCT GCT GGA AGG TGC TT-3'. A control experiment without cDNA was included for each PCR mix. Specific primer pairs (20 bp) were designed for the 12 TDFs chosen for validation, using the Primer 3 software (Additional File 5). Data were analyzed using MxPro QPCR software (Stratagene). The C_t _(cycle at which the increase of fluorescence exceeded the threshold setting) was used to calculate the fold changes (FC) in each infected sample compared to the expression level detected in the corresponding sample under control conditions (baseline) with the following formula: FC = 2 ^-ΔΔCt ^where ΔΔC_t _= (C_t target _- C_t actin_) _infected sample _- (C_t target _- C_t actin_) _uninfected sample_.

Evaluation of expression of FOM genes was carried out by calculating the difference between the Ct of the gene analyzed and the Ct of melon actin (AY859055), used as a normalizer.

## Authors' contributions

SS participated in the scoring of the c DNA-AFLP bands, performed blast and database homology search and the assignment of the functional categories. The author performed the real time experiments and collaborated in writing the manuscript and in preparing table and figures. AP participated in interpretation and discussion of cDNA-AFLP data and wrote most of the manuscript in particular all parts related to plant responses to infections. The author also collaborated in preparing table and figures. LL performed the infections and the reisolations from inoculated plants, collected and processed infection data, performed the extraction of RNA from fungal colonies, and checked the autoradiographic films for profile clustering. AF performed all blast and database homology searches and profile clustering. MS performed the statistical analysis and the description of the different patterns of the host colonization by the three pathogenic strains of FOM. MD participated in conceiving the study and to produce the cDNA-AFLP data.

JH performed DNA and RNA extraction from melon plants, participated in cDNA-AFLP and in real-time RT-PCR experiments. NF prepared and provided all the genetic material used in this work and participated in the interpretation and discussion of cDNA-AFLP data. AB is the research coordinator, conceived the study, participated in all steps of the analysis and extensively collaborated in writing the manuscript, in particular on aspects related to fungal infection process, vascular colonization and molecular basis of pathogenicity. All authors read and approved the final manuscript.

## Supplementary Material

Additional file 1 **Complete list of melon genes modulated by infection with avirulent (ISPaVe1070 race1) or virulent (ISPaVe1018 and ISPaVe1083 strains of race 1,2) strains of *Fusarium oxysporum *f. sp. *melonis*, organized by clusters as defined in the results section**. Identification numbers (ID) correspond to the progressive numbering of bands in the gels. The primer combination used to visualize each band (Bst-Mse column) and the corresponding length of the fragment are reported, along with the accession number retrieved from the Melon Unigene or Uniprot database, the annotation, the corresponding blast score, and the functional category to which each transcript was assigned. In addition, the file reports the expression profile of each transcript, estimated on the basis of the band intensity with a score ranging from -3 to 3, in comparison to the corresponding band in the mock-inoculated controls. Click here for file

Additional file 2 **Complete list of *Fusarium oxysporum *f. sp. *melonis *(FOM) transcripts identified in infected melon plants, either specifically *in planta*, or both *in planta *and *in vitro***. Identification numbers (ID) correspond to the progressive numbering of bands in the gels. The primer combination used to visualize each band (Bst-Mse column) and the corresponding length of the fragment are reported, along with the accession number retrieved from the database, the annotation, the corresponding blast score, and the functional category to which each transcript was assigned. In addition, the file reports the expression profile of each transcript, in each interaction and at each time point, estimated on the basis of the band intensity with a score ranging from 1 to 3. Click here for file

Additional file 3 **List of the core 54 melon genes, which remain modulated in a coherent way (induced or repressed) throughout the experiment (from 4 dpi onwards) in the incompatible interaction between melon and the FOM race 1 strain ISPaVe1070**. Click here for file

Additional file 4 **Complete list of *Fusarium oxysporum *f. sp. *melonis *(FOM) transcripts identified by cDNA-AFLP analysis from fungal samples of colonies grown *in vitro *and selected for sequencing**. TDFs were chosen on the basis of their differential abundance in the different FOM strains. Identification numbers (ID) correspond to the progressive numbering of bands in the gels. The primer combination used to visualize each band (Bst-Mse column) and the corresponding length of the fragment are reported, along with the accession number retrieved from the database and the corresponding blast score and annotation. In addition, the file reports the presence (+) or absence (-) of expression of each transcript for the 3 strains of FOM. Click here for file

Additional file 5 **List of primers used in Real time RT-PCR analysis**. Identification numbers (ID) correspond to the progressive numbering of bands in the gels. The accession codes for each gene represent the corresponding database in which homology was identified, MU = Melon Unigene, FOX = Fusarium Comparative Database. Click here for file
